# Design and Development of Spray-Dried Microsystems to Improve Technological and Functional Properties of Bioactive Compounds from Hazelnut Shells

**DOI:** 10.3390/molecules25061273

**Published:** 2020-03-11

**Authors:** Tiziana Esposito, Teresa Mencherini, Pasquale Del Gaudio, Giulia Auriemma, Silvia Franceschelli, Patrizia Picerno, Rita P. Aquino, Francesca Sansone

**Affiliations:** Department of Pharmacy, University of Salerno, Via Giovanni Paolo II, 132, 84084 Fisciano (SA), Italy; tesposito@unisa.it (T.E.); pdelgaudio@unisa.it (P.D.G.); gauriemma@unisa.it (G.A.); sfranceschelli@unisa.it (S.F.); ppicerno@unisa.it (P.P.); aquinorp@unisa.it (R.P.A.)

**Keywords:** hazelnut shells by-product extract, spray-dried microsystems, multicomponent-based matrix, long-term stability, improvement of the chemopreventive effect

## Abstract

An extract obtained from hazelnut shells by-products (HSE) has antioxidant and chemopreventive effects on human melanoma and cervical cancer cell lines, inducing apoptosis by caspase-3 activation. A clinical translation is limited by poor water solubility and low bioavailability. Dried plant extracts often show critical characteristics such as sticky/gummy appearance, unpleasant smell, and instability involving practical difficulties in processing for industrial use. A spray drying method has been applied to transform raw HSE in a microparticulate powder. The biopolymeric matrix was based on l-proline as loading carrier, hydroxyethylcellulose in combination with pectin as coating polymers; lecithin and ethanol were used as solubility enhancers. A Hot-Cold-Hot method was selected to prepare the liquid feed. The thus prepared powder showed good technological properties (solid-state, particle dimensions, morphology, and water dissolution rate), stability, and unchanged chemopreventive effects with respect to the unprocessed HSE.

## 1. Introduction

By-products of agro-food manufacturing chains contain polysaccharides, proteins, vitamins, fats, and phytochemicals. The recovery of bioactive components with health-promoting benefits suggests the exploitation of vegetable residues as functional ingredients in developing nutraceuticals, functional or novel foods, and pharmaceutical products [1,2,3]. Today, there is an increasing interest in the use of nutraceuticals, based on plant extracts or food by-products, as potential chemopreventive agents, to be used in combination with chemotherapeutics, as a new strategy in cancer control [4,5,6]. In previous work, we have produced a polyphenol-rich extract (HSE) from the hazelnut shells, major by-product, together with skins [7] of the kernel industrial processing. The neolignan lawsonicin was identified as the HSE chemical marker. Moreover, the extract antioxidant and inhibitory effect inducing apoptosis on human melanoma and cervical cancer cell growth were demonstrated [8]. Due to its functional properties, the production of HSE represents an efficient way to re-use and up-cycle hazelnut shells, turning these by-products, destined for landfill, into a resource of human health-promoting molecules. However, as all dry plant derivatives, HSE appears as crystalline sticky material, not completely soluble in water and aqueous biological fluids [9,10]. These critical features could limit the real use of natural polyphenol derivatives as therapeutic/nutraceutical agent, negatively affecting the absorption process, the bioavailability after the intake, the handling, and the storage during the industrial scale use [11]. Moreover, the potential phytochemicals degradation process over time may reduce the active ingredient content and, consequently, the functional value of the plant extract [10,12,13]. In the present research, a particle engineering approach, involving the production of microparticles by spray drying technique, is proposed to overcome the stability and bioavailability problems, converting the raw HSE in a powder, suitable for the final administration in various dosage forms (mainly oral or topical ones) as an active ingredient [14,15].

The spray drying is an atomization technique, widely employed in the food and pharmaceutical field, to transform a starting liquid feed into a microparticulate powder [16]. This microencapsulation technique has been successfully applied to heat-sensitive materials such as plant extracts to obtain a processed material showing enhanced technological properties (solid-state, particle dimensions, morphology, and water dissolution rate), stability of active components within the polymeric matrix, and an efficient release to target site of application/action, with respect to the plant raw extract [13,17,18,19,20]. The design and development of plant-based powders are a technological challenge involving the study of several critical aspects (solvent system, liquid feed preparation method, polymeric matrix components) and the optimization of the experimental plan also considering both the chemical-functional characteristics of the plant extract and the intended use of the final product.

The combination of natural products can make it possible to obtain biomaterials with new potential biological functions. In this sense, the association of plant extracts with functional carriers may offer a promising strategy for obtaining new drugs [21].

Amino acids (arginine, phenylalanine, and leucine) are often used in the microencapsulation process as effective dispersibility enhancers able to load active ingredients [22,23,24]. Among amino acids, l-proline (P) possesses the highest solubility in water and alcohol, and it has been proven to be essential to produce collagen and healthy joints, tendons, and skin [25]. Some research reported the multifunctionality of P showing a protective role against cellular osmotic and oxidative stress, protein chaperoning, cell signaling, programmed cell death, and extracellular matrix (ECM) integrity. As ECM degradation plays a critical role in the formation of tumors and metastasis, P seems to present an added value as a loading polymer of chemopreventive ingredients [26,27]. P metabolism affects cell survival and death outcomes by influencing the intracellular redox environment during oxidative and nutritional stress conditions. Moreover, the proline oxidative catabolism induced apoptosis through the mitochondrial (intrinsic) pathway [27,28,29]. Furthermore, P acts as an antioxidant by upregulation and stabilization of antioxidant enzymes, scavenging of ROS, metal chelation, the equilibrium of intracellular redox homeostasis, and improvement of cellular resistance to hydrogen peroxide [30]. There is no reported information on P as a carrier for encapsulation of functional natural extract. Moreover, this is the first microencapsulation study focused on the encapsulation efficiency and in vitro biological activity of hazelnut shells extract loaded in microsystems.

Despite high functionality and technological versatility, the P high crystalline degree makes it difficult to use for microparticles production countering the amorphization phase during the transformation process [31]. The main protective role of microencapsulation technology is to form a membrane/shell (wall material) around particles or droplets of active ingredients that are being encapsulated (core material) [10]. Thus, in this work to obtain a satisfactory spray-dried product, the use of hydroxyethylcellulose (HEC) as coating and stabilizing agent during the microencapsulation process by spray drying was necessary. HEC is a non-ionic, biocompatible, non-toxic polymer (average molar mass of about 120.000 Da) derived from cellulose [32]. The HEC high water solubility and stability in the pH range of 3.0 to 11.0 make easy its combination with saline solutions also in the presence of metal ions [33]. HEC is a versatile pharmaceutical excipient with many applications, as tablet binder, modified-release matrix former, film-forming agent, and thickening agent. The mechanical properties of HEC gels are also useful in the design of topical formulations, making easy the removal from the tube, the application and retention on the skin, and the feel of the product after application [34].

A good encapsulating agent (wall material) should have emulsifying and film-forming properties, display low hygroscopicity, have low viscosity at high solid contents, be biodegradable, soluble in aqueous solvents, bland in flavor/tasteless, low-cost, non-toxic, and food-grade [10]. However, no single encapsulant possesses all these properties, and because of that, two or more encapsulants must often be used in combinations. Pectin (PEC) is a structural component of the vegetable cell wall. It is recovered from higher plants and widely used as a gelling agent, stabilizer, and thickener in foods (E440). The polymer viscosity depends on the molecular weight, pH, ionic strength, and arrangement of carboxyl groups [35,36]. PEC is a heterogeneous polysaccharide made up of linear (1→4)-linked-α-d-galacturonic acid residues partially broken off by (1→2)-linked side chain consisting of l-rhamnose and some other neutral sugar residues [37]. Pectin can be classified in low methoxylated pectin (DE < 50%) and high methoxylated pectin (DE > 50%) according to their degree of esterification (DE), expressed as a percentage of carboxyl groups [36,38]. High methoxylated pectin (DE > 50%) form a particularly stable gel polymeric network, both at acid pH (pH ≈ 5) and in the presence of sugars, without adding divalent ions as occurs for pectin with a low DE or alginates. Moreover, the high methoxylated pectin is characterized by a low hygroscopicity; an important property to improve product stability during storage [39].

The lecithin (L) molecule is a food additive (E322) used as an excellent natural emulsifier, stabilizer, and dispersing agent. The amphiphilic properties allow them to increase the water affinity of non-polar ingredients (fats), arranging the hydrophobic portion around them, and the hydrophilic moiety in the aqueous phase [40].

The aim of the present research was the production of new polymeric microparticulate HSE-based powders by spray drying technique. The appropriate polymeric matrix, as coating polymers and loading carriers, able to transform HSE in a microparticulate powder form, was selected. The influence of instrumental and operating parameters on yield and encapsulation efficiency was evaluated. The produced microparticulate powders were characterized for solid-state (particle dimensions, morphology, amorphous or crystalline form), and water dissolution rate. The optimized formulation was subjected to accelerated stability tests under harsh storage conditions (ICH-International Conference on Harmonization). Finally, the functional stability of the HSE-loaded particle system was investigated.

## 2. Results and Discussion

### 2.1. Microencapsulation Process, Morphological, and Dimensional Characterization

The technological studies start with the design and development of a tandem system polymeric matrix processable by spray drying technique. The matrix system was designed to be able to both efficiently load the polar extract HSE and transform it into a stable, functional, microparticulate powder useful as a therapeutic ingredient.

The viscosity was a limiting parameter to process a liquid feed by the spray drying technique [16,41]. A too high viscosity could obstruct the atomizer nozzle, also causing incomplete solvent evaporation, and low process yields, which negatively affected the particle morphology [35]. Thus, a series of pilot experiments were conducted to determine the appropriate HEC concentration giving a liquid feed compatible in viscosity with the spray drying process. Among the applied HEC concentrations (between 0.1% and 1.0%, *w*/*v*) the best instrumental processability was obtained using the 0.1% and 0.2% (*w*/*v*) concentration of HEC (relative viscosity 68.31 cP and 75.56 cP, respectively). More than 45 pilot experiments (blank and loaded powders, Table 1) were carried out to find the optimum of liquid feed in terms of polymer concentrations and weight polymer ratios with respect to the process yields, loading ability, and resultant powders technological characteristics. As an example, only three batches (3,9,12) of blank powders and two batches of loaded (13,15) ones have been selected, discussed in the text, and reported in the Table 1 as demonstrative samples of the taken experimental steps. Additional information was included in the Supplementary Materials.

**Figure 1:**
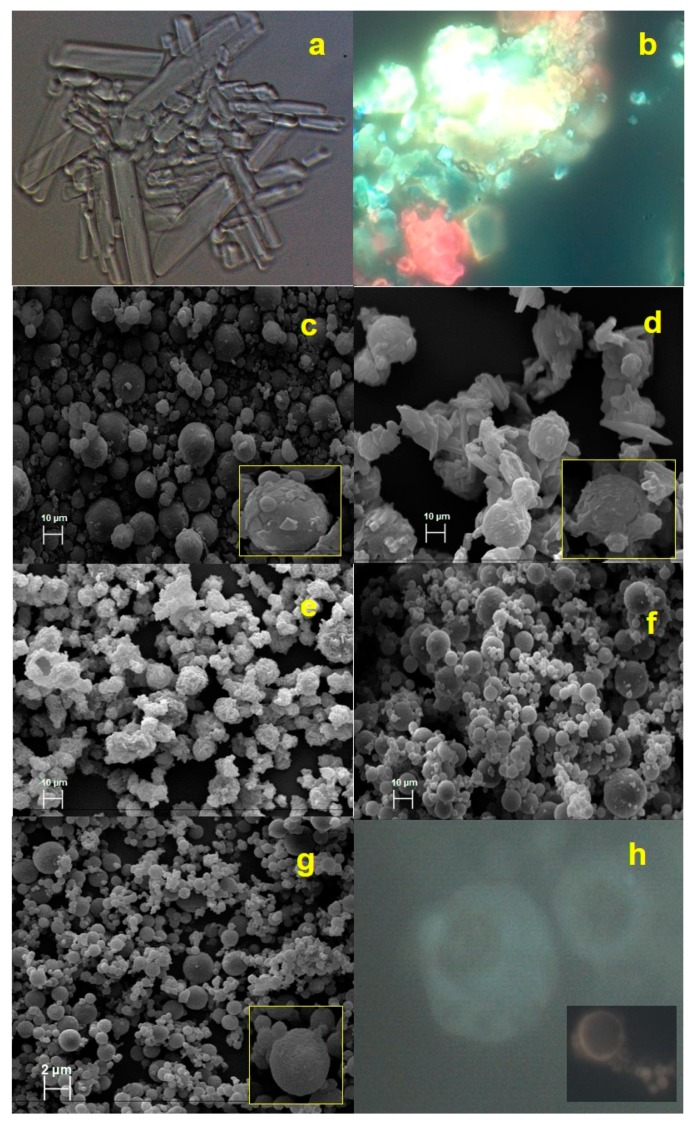
Fluorescence (FM) images in bright field (**a**) P, proline spray drying, (**b**) hazelnut shells by-products (HSE) raw material in crystalline form; scanning electron microscopy (SEM) micrographs (**c**) Batch-3; (**d**) Batch-13, (**e**) Batch-9, (**f**) Batch-12, (**g**) Batch-15, FM image (**h**) Batch-15.

Preliminary satisfactory results in terms of matrix processability were obtained with an HEC and P concentration of 0.2% and 5.0% *w*/*v* (Batch-3), respectively. Normally, the morphology of P shows a needle crystalline form [25] (Figure 1a) and the unprocessed extract appeared as a material in a cluster form with irregular shape and surface (Figure 1b) and exploiting red and yellow fluorescence due to the different nature of polyphenols present in the extract (Figure 1b).

Spray-dried Batch-3 showed a P interaction with HEC, involving in a spherical agglomeration process, without totally losing its crystalline state (Figure 1c) [42] causing fractures and clusters on particles surface. Moreover, the loading with HSE further negatively affected the agglomeration process of the matrix system (Batch 13, Figure 1d). After the spray drying process, P, in combination with HEC and HSE, did not lose its crystalline state, leading to an irregular and not homogeneous solid-state Batch 13, Figure 1d).

With the aim to both promote the particle formation and reduce the crystalline surface, also increasing process yield, high methoxylated pectin from citrus (PEC) was selected as an additional coating polymer, separately included in the basic polymeric matrix. The presence of crystals can fracture the outer surface of the particles and promote the release of the extract. This is a cause of instability because the extract was exposed and, therefore, subject to degradation. In previous work, PEC has proved suitable to stabilize maltodextrins as a carrier of sticky plant extracts [35]. The percentage of PEC was tested in formulation from 0.25 to 1% *w*/*v*. The resultant strategy was best effective (yield 45.0%) at the 0.5% PEC concentration (Batch-9, Figure 1e, Table 1). The highest concentrations (1.0% *w*/*v*) of PEC, in combination with HEC, led to a drastic reduction in process yield (15.6%), probably caused by an increase in the viscosity of the matrix. As shown in Figure 1e, the addition of PEC as coating copolymer improved the spherization of the particles, and no aggregates were detected.

Once the polymeric matrix conditions were established, the next step was to load the HSE extract. Lecithin (L) as emulsifier dissolved in ethanol (EtOH) [43] was added to the liquid feed preparation to overcome the critical solubility characteristics of the plant extract. Moreover, a “Hot-Cold-Hot” (H-C-H) method preparation was used. The formulation of a multi-component matrix requires, for reasons linked to the different characteristics of the ingredients, a multi-step preparation method. The Hot-Cold-Hot method depends on the solubilization conditions of HEC that need heating to dissolve in water. Subsequently, the extract needs a lower temperature because it is a thermosensitive substance. The thus prepared liquid feed reflected in the best-obtained result in terms of process yield (50%) and particle morphology (Batch-12, Figure 1f) for unloaded powders. The best HSE concentration, compatible with the developed polymeric system, resulted in the 0.25% *w*/*v* (Batch-15, yield 43.0%, Table 1), with respect 0.50% *w*/*v* (Batch-13, yield 39.8%, Table 1).

The actual extract content (AEC-HSE) of Batch-15 resulted very close to the amount of extract used to prepare the liquid feed (theoretical extract content, TEC-HSE). Consequently, the loading efficiency (LE) value, calculated as the ratio of AEC to TEC, was very satisfying (95.12%, Table 1). However, for optimized powders, the process yield did not exceed 50% probably for the low amount of material sprayed (100 mL) and the loss of the smallest and lightest particles with the exhaust of the spray dryer [15].

Morphological analysis showed that for Batch-15 (Figure 1g), spherical, well-formed, and completely coated microparticles had been obtained during the spray drying process. Furthermore, the extract was homogeneously distributed and better encapsulated within the microparticles (Figure 1g,h). In fact, Batch-15 microparticles showed a pale-yellow fluorescent (Figure 1h), which was the result of the combination between red/yellow-HSE and blue blank powder as evidence of interaction in forming a homogeneous matrix.

As reported in Table 1, Batch-13 and Batch-15 microparticles also had different size distribution (d_50_ 18.41 and 3.0 µm) with the lowest mean diameter observed for Batch-15 (Table 1). This difference was due to the right physical interaction between matrix components leading to the rearrangement of P crystals in spherical agglomerates during the spray drying process. The use of the organic lecithin solution to better expose the extract to the aqueous polymeric feed seemed to positively affect the interaction of extract with the matrix components, resulting in the reduced dimensional distribution of the obtained particles.

### 2.2. Thermal Analyses

**Figure 2:**
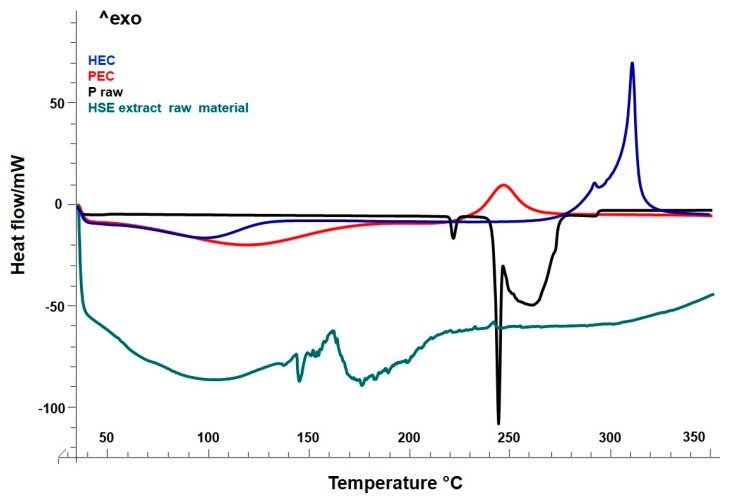
Differential scanning calorimetry (DSC) of raw materials: HEC, blue line; PEC, red line; P, black line; HSE extract raw material, green line.

**Figure 3:**
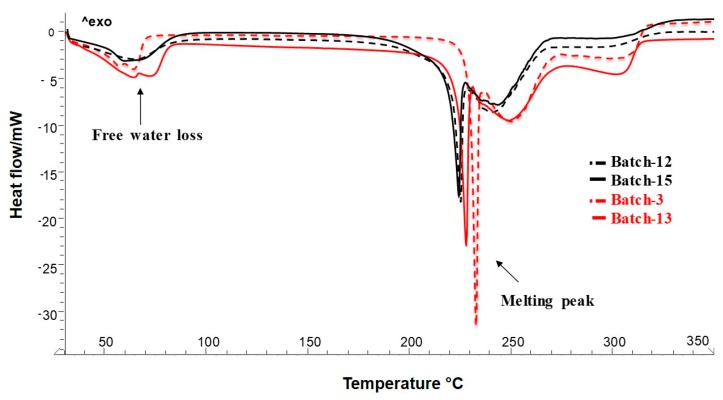
Differential scanning calorimetry (DSC) of Batch-12, Batch-15, Batch-3, and Batch-13.

To provide information on solid-state and extract-polymer interactions, as well as on the physical stability of materials after the technological process, differential scanning calorimetric technique was used [44]. To evaluate the physical interaction after the formation of the particles during the spray drying process, the thermal trends by DSC of all raw materials with respect to the spray-dried formulations were shown (Figure 2 and Figure 3).

The heating curve of the HEC raw material showed a slight endothermic event at the baseline above 100–120 °C, due to the dehydration phase. The water loss up to 120 °C was generally followed by a two-step thermal event of cellulose, depolymerization, and decomposition [45]. In the thermogram (Figure 2, blue line), only an exothermic transition was visible (at around 310 °C), which may be related to the depolymerization of cellulose material. This event anticipated the pyrolytic decomposition [46], which could occur in a 400 to 470 °C range of temperature, and was not detected here. The proline raw material showed a first endothermic peak at 217 °C with the final melting peak at 246 °C (Figure 2, black line).

The thermal profile by DSC of the unprocessed HSE raw extract (Figure 2, green line) exhibited a series of endothermic events due to the melting of the active components, mainly polyphenols, in a range of temperatures between 130 and 250 °C. As for other natural extracts, it was not possible, for HSE, to see a single melting peak as for pure components [17,18,35].

Figure 3 shows the thermal behavior of the spray-dried powders. The first endothermic event, at baseline, in the temperature range of 50 to 100 °C, was due to free water loss. As shown by the thermogram (Figure 3), the amount of residual water was lower for Batch-15 with respect to Batch-3, and Batch-13 could positively affect the stability of Batch-15 powder.

Comparing DSC thermal profiles of Batch-12 and Batch-3 (blank powders, Figure 3, dotted black and red line, respectively), proline showed an anticipation of melting peak probably due to the effect of agglomeration on P crystals [31,42], shifted by 8 Celsius degrees in Batch-15 (221.45 °C) with respect to Batch-13 (228.81 °C). This event was accompanied by a reduction of enthalpy, indicating a reduction in crystallinity. Even if there was a melting peak attributable to a crystalline residue of proline, the produced systems could be defined as an amorphous multicomponent ingredient. The structure of amorphous solids was not random at the molecular level but may possess short-range order, residual crystallinity, polymorphic states, and regions of different density. Many pharmaceutical formulations (multi-component systems) are formed by either single or multiple active substances and drug excipients. One or more of the components can be present in the amorphous state [47].

Finally, in all thermograms, it was possible to observe a thermal event around 290 °C, due to the final product degradation. This event possessed a lower intensity for Batch-15 (Figure 3) for Batch-13 (Figure 3), indicating that the extract was completely encapsulated in the matrix. No new peaks ascribable to chemical interactions were detected.

The thermal analysis of the optimized formulation Batch-15 confirmed that extract well interacts in forming microparticles with the polymeric matrix. Moreover, DSC confirmed SEM (Figure 1g) results that showed microparticles well-formed with a reduction of proline in a crystalline state, also supposing a consequent better behavior instability [20,35].

### 2.3. In Vitro Dissolution/Release Tests

**Figure 4:**
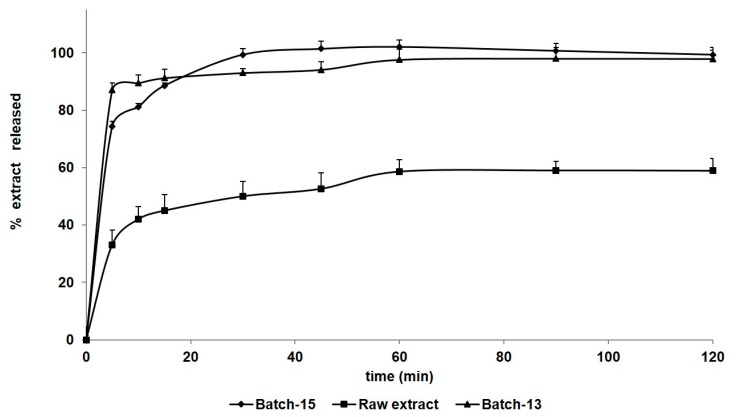
Dissolution/release profile of Batch-13 and Batch-15 microparticles compared with HSE (unprocessed extract).

The water dissolution/release profile of Batch-13 and Batch-15 microparticles was improved, showing a doubled release of HSE for the Batch-15 with respect to the raw material (Figure 4). In 15 min, 88.5% and 91.2% of the release/dissolution of the extract was obtained from Batch-15 and Batch-13, respectively. At the same time, only 33.12% of the unprocessed extract (HSE) was dissolved. This result can be explained by an increase of the microparticles-water interaction due to both the smallest dimensions (d_50_ 3.02 and 18.41 µm) and amorphous state of the particles [44]. The improved HSE/matrix interaction, combined with the use of lecithin and further dimensional reduction, promotes the release of HSE from Batch-15 compared to Batch-13, to achieve 100% dissolution in 30 min. The very low standard deviation obtained confirms the homogeneity of the formulation also in terms of loading and distribution of the extract in the matrix. The increase of solubility in aqueous media is of great importance because this characteristic can enhance the functionality of the spray-dried powders [9,13].

### 2.4. Stability Studies of Batch-15

**Figure 5:**
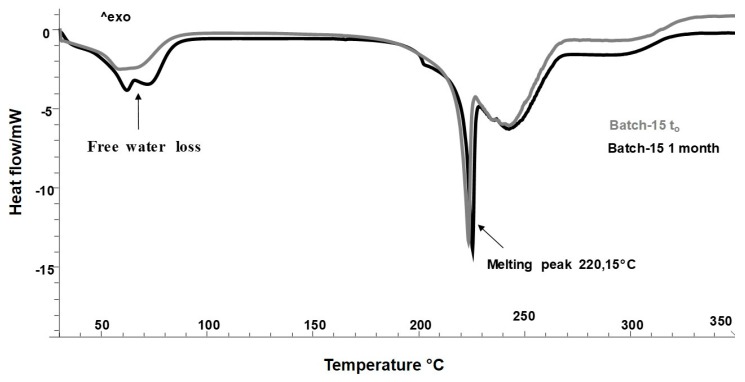
Differential scanning calorimetry (DSC) thermograms of Batch-15 (48 h in a desiccator) at *t*_0,_ and after 1 month.

Based on the results obtained, Batch-15 proved to be the best formulation in terms of process yield, encapsulation efficiency, thermal profile, and the improved release of the functional compounds from the extract in water. The extract, as previously demonstrated, contains a high polyphenols content. The polyphenols possess several biological activities, such as antioxidant, antimicrobial, anti-inflammatory, anti-cancer, with beneficial effects on human health [8]. However, some of them (phenolic acids, flavonoids, neo-lignans), if exposed to environmental stress, are unstable, because they could undergo oxidation and degradation processes, which take place especially during the storage period [48]. This causes a reduction of active compounds, and consequently, a lower nutritional and biological value [3]. In order to evaluate the shelf life and the effect of the spray drying process on the stability of HSE under storage conditions, accelerated stability test was performed according to the ICH guidelines (International Conference Harmonization), under harsh storage condition, for 6 months, at 40 °C and 75% Relative Humidity (RH%). Microscopy and DSC analyses were used to evaluate changes in structure and possible degradations. Before further characterizations, Batch-15 has been stored for 48h in a hermetically sealed desiccator (25 °C), in order to evaporate the residual process humidity content. Figure 5 shows the thermal profiles of Batch-15 after a storage period of 1 month (black line) with respect to Batch-15 at *t*_0_ (48h in a desiccator, gray line). The results displayed the presence of a small percentage of free water, absorbed during the spray drying process, after 48 h in a desiccator. The peak was already visible after 1 month (Figure 5, gray and black lines). However, the presence of water, only affected the morphology of particles without causing no degradation events or formation of chemical interactions (Figure 5) because no new peaks have been detected.

**Figure 6:**
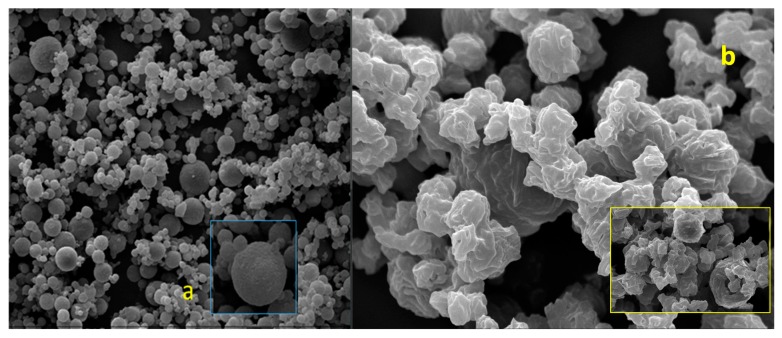
Scanning electron microscopy (SEM) images of Batch-15 (48 h in a desiccator) at *t*_0_ (**a**) and after 1 month (**b**).

In fact, in the SEM images, no breakings were visible in the microstructures, although a high degree of aggregates and particles showed an irregular shape and surface with the predominant crystalline aspect evident (Figure 6 and b).

The physical instability was probably due to the residual process humidity content of the powder. Hygroscopicity results indicated that during the harsh storage conditions, a water loss occurs, gravimetrically determined, of 2.40 g/100 g ± 0.82. The mobility of this amount of residual free-water was responsible for morphology changes.

**Figure 7:**
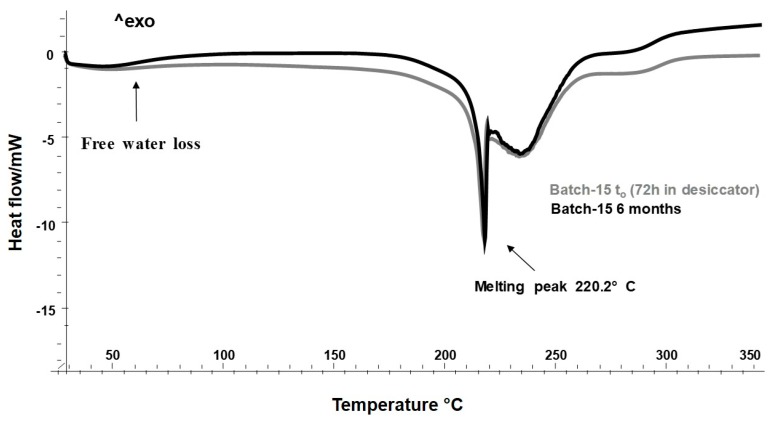
Differential scanning calorimetry (DSC) thermograms of Batch-15 (72 h in a desiccator) at *t*_0,_ and after 6 months.

**Figure 8:**
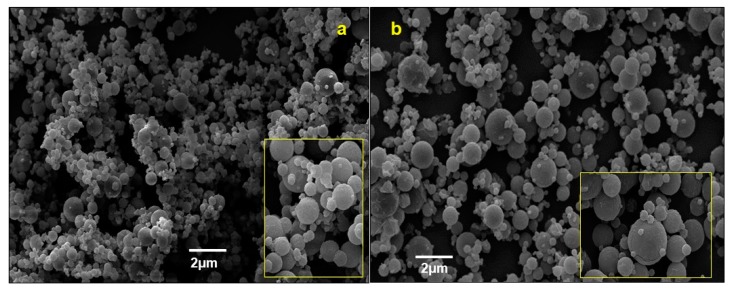
Scanning electron microscopy (SEM) images of Batch-15 (72 h in a desiccator) at *t*_0_ (**a**) and after 6 months (**b**).

Thus, the storage period of Batch-15 in desiccator was extended to 72 h, and the accelerated stability assay was repeated, in the same condition. The results obtained exhibited a clear reduction of the free water (gravimetrically quantified, 0.41 g/100g ± 0.03) (Figure 7), and the morphology of the particles resulted unaltered, also after 6 months (Figure 8 a,b).

The lawsonicin content, the major compound of HSE, and the free radical scavenging activity were verified, both after the spray drying process (after 72 h in the desiccator) and after 6 months under the storage condition and compared with the unprocessed extract (HSE). The results, summarized in Table 2, showed that Batch-15 did not exhibit a significant reduction of the lawsonicin content (lower than 1%) after the transformation process and 6 months of storage. On the contrary, HSE proved significantly (*p* < 0.05) lower active content. Moreover, the antiradical activity was evaluated by the DPPH test to verify that the formulation process and the storage period have not altered the free radical scavenging activity of HSE [35]. As reported in Table 2, the effect remained unaltered after the spray drying process (EC_50_ 33.20 ± 0.61 µg/mL) and until 6 months of harsh storage conditions (EC_50_ 32.80 ± 1.01 µg/mL). On the contrary, at the same conditions, HSE raw extracts proved a significant (*p* < 0.05) reduction of EC_50_ values from 33.42 ± 1.40 µg/mL to 40.04 ± 2.11 µg/mL.

The results demonstrated that the optimized matrix and process parameters allowed to protect the antiradical efficacy of HSE, obtaining a long-lasting, stable microparticulate system.

### 2.5. Functional Activity of Batch-15

As demonstrated previously, HSE possesses not only a significant scavenging activity in vitro against the radical DPPH but also an inhibitory effect on human melanoma and cervical cancer cell lines. To investigate if the spray drying process affects HSE functional properties, the inhibitory effect of the optimized batch (Batch-15) on the growth of human normal (HaCaT) and cancer (A375, SK-Mel-28, and Hela) cell lines was evaluated. The results of the MTT test (Table 3) showed that the inhibitory effect of Batch-15 (AEC% 3.9%, *w*/*w*) remained unchanged for SK-Mel-28 (IC_50_ 10.550 mg/mL containing 0.411 mg/mL of HSE) or resulted in improved for A375 and HeLa (IC_50_ 4.550 and 8.070 mg/mL, containing 0.177 and 0.315 mg/mL of HSE, respectively), proving as the spray drying process did not affect HSE efficacy. On the contrary, the produced amorphous microsystems were able to improve the wettability of HSE and the release of HSE active components in water cellular environmental [13]. Moreover, the occurrence of proline in the multi-polymeric Batch-15 matrix will affect the cytotoxic properties of the HSE raw extract. An adequate supply of amino acids, such as proline, allows an adequate formation and structural stability of the extracellular matrix (ECM). The microenvironment of ECM plays an important role in several phenomena occurring within the cell. Alterations in its structure, determined by changes in the molecular composition, can destroy the microenvironment homeostasis, causing the formation of tumors, and metastases [49,50,51]. In particular, the proline metabolism, generated by the proline oxidase (PRODH), seems to be able to catalyze the generation of Reactive Oxygen Species (ROS), usually produced in high levels in cancer cells [52], and to induce apoptosis in several tumor cell lines [50]. Moreover, PRODH helps to maintain the ATP levels of the cell during oxidative and nutritional stress conditions, because changes in the intracellular redox environment are responsible for cell survival and death [26,50]. Finally, the IC_50_ of Batch-15 resulted > 15 mg/mL on a normal skin cell line (HaCaT) used in the assay, supporting its safe use at active concentrations in cancer cells (from 8.070 to 10.550 mg/mL). The results suggest that the developed spray-drying method is suitable to transform HSE raw material in a water-soluble, easy handling, and functional powder that can be used as in topical or oral dosage forms, useful as an adjuvant in cancer forms treatment or prevention.

## 3. Materials and Methods

### 3.1. Chemicals

Analytical grade methanol (MeOH), HPLC-grade methanol (MeOH), isopropanol, ethanol (EtOH), 1,1-diphenyl-2-picrylhydrazyl radical (DPPH), α-tocopherol, and l-proline (P) were obtained from Sigma-Aldrich (Milan, Lombardia, Italy). Medium viscosity hydroxyethylcellulose (HEC, Natrosol MR) and lecithin (L, E322) were supplied by ACEF Spa (Fiorenzuola D’Arda, PC, Italy). Pectin (PEC 09/051, 76% degree of esterification) was recovered from the citrus peel obtained during the processing of Agrumi Gel s.n.c. Company (Barcellona Pozzo DI Gotto, Messina, Italy). HPLC-grade water (18 mΩ) was prepared by a Milli-Q50 purification system (Millipore Corp., Bedford, MA, USA). The hazelnut shells extract (HSE) was prepared with the extraction method previously reported [8].

### 3.2. Liquid Feeds Preparation and Spray Drying Conditions

Before spray drying, the viscosity measurements of liquid feeds were carried out by a Visco Basic plus viscometer (FungiLab s.a., Barcelona, Spain) at 25 °C, with a Spindle L_1_ between 0.3 and 100 rpm, every 60 s after the rotation speed started. The results were expressed as centiPoise (cP), and the accuracy of the measurement was expressed in terms of instrumental reliability value (valid range 18 to 100).

Preparation method used:

- Hot-Cold-Hot method (H-C-H): The liquid feed was produced using 0.2% *w*/*v* HEC, 5% *w*/*v* P and 0.2% L at 85.0:3.4:8.2:3.4 P:HEC:PEC:L ratio (total amount 6.15 g). PEC was added in 80 mL of water at 75 °C; at room temperature P was included, and finally, at 50 °C, HEC was dissolved, leaving under stirring overnight. Separately, 0.25% or 0.50% *w*/*v* of HSE was dissolved adding 20 mL of ethanol and 20 mL of a 1% *w*/*v* L solution by homogenization with an Ultra-Turrax T-25 (IKA ULTRA-TURRAX T25 digital) at 10,000 RPM for 5 min. The suspension containing the extract (HSE) was slowly poured into the feed under continuous magnetic stirring.

All the liquid feeds were spray-dried using a Büchi B-191 Mini Spray Dryer (Büchi Laboratoriums-Technik, Flawil, Switzerland). The Mini Spray Dryer operates according to a co-current drying gas (e. g. air in open mode) and product stream. This means that sprayed products and hot gas have the same flow direction from downward.

The experimental conditions were drying airflow 600 L/h, air pressure 6.5 bar, aspirator 100% (maximum gas flow rate of about 35 m^3^/h), inlet/outlet temperatures 125/85 °C, Ø nozzle = 0.7 mm, spray flow feed rate 3.8 mL/min.

Each preparation was produced in triplicate. Before characterization, the powders were stored in the desiccator (48–72 h).

### 3.3. Powders Characterization

#### 3.3.1. Yield and Loading Efficiency

A gravimetric method (balance Crystal 100 CAL—Gibertini Novate, Milanese, Italy, max 110 g d = 0.1 mg; +15 °C/30 °C) was used to determine production yield, and the result was expressed as the weight percentage of the final product compared to the total amount of the materials present in the liquid feed.

The Theoretical Extract Content (TEC) was calculated as the percentage of extract (HSE) content compared to the initial total amount of all feed components before spray drying.

Actual Active Content (AAC) of the unprocessed extract HSE and spray-dried microparticles (Batches 13 and 15) was determined by HPLC-DAD method, according to [8], and expressed as the lawsonicin content percentage to 100 mg of powder.

The Actual Extract Content (AEC) was derived by AAC and calculated as:

AEC% = AAC_Batch 13/15_/AAC unprocessed HSE × 100 (1)

The extract encapsulation efficiency (EE%) was the ratio of the actual to the theoretical extract content: [13]:

EE% = AEC/TEC × 100(2)

Each analysis was made in triplicate and results expressed as an average value.

#### 3.3.2. Quantitative Analysis by HPLC Method

30 mg of each batch was dissolved in 10 mL of mixture methanol and distilled water (8:2) and sonicated for 20 min. The lawsonicin (HSE chemical marker) content was determined by an HPLC apparatus (Agilent 1100 series system equipped with a Model G-1312 pump, a Rheodyne Model G-1322A loop (20 μL), a DAD G-1315 detector, and a Nucleodur 100-5 C_18_ column (150 × 4.6 mm, 5 μm, Machery-Nagel). An Agilent integrator was employed to calculate peaks area. The analysis was carried out with solvents, elution gradient, and experimental conditions previously applied [8].

#### 3.3.3. Particle-Size Analyses

A Laser Light Scattering (LLS) granulometer (Beckman Coulter LS 230, Particle Volume Module Plus, Brea, CA, USA) was used to evaluate the dimensions of the raw materials and all the batches obtained by spray drying. Isopropanol was employed to suspend the powders, and 150 µL of each sample was introduced into the analysis cell to obtain an obscuration between 8% and 12%. The mathematical Fraunhofer model was applied to calculate particle size distribution. The analyses were conducted in triplicate: The results were expressed as d_50_, indicating the volume diameter at the 50th percentile of the particle size distribution. The span was determined as [(d_90_ − d_10_) / d_50_]; a not homogeneous particle size distribution was correlated to a higher span value (>3) [20].

#### 3.3.4. Morphological Analyses

The morphologies of raw materials and produced particles were analyzed via scanning electron microscopy (SEM) using a Carl Zeiss EVO MA 10 microscope operating at 17 kV; the powders were coated with Au/Pd and eventually observed at different extensions.

The optical microscopy assays were performed in fluorescence and brightfield mode observing the samples with a Zeiss Axiophot microscope with optical mode, equipped with 40, 63, and 100 × 1.4 NA plan Apochromat oil immersion objectives (Carl Zeiss Vision, München-Hallbergmoos, Germany). For the fluorescence mode, a standard DAPI (40,6-diamidino-2-phenylindole) optics that adsorbs violet radiation (max 372 nm) and emits a blue fluorescence (max 456) was used.

#### 3.3.5. Differential Scanning Calorimetry (DSC)

Differential Scanning Calorimetry on an indium calibrated Mettler Toledo DSC 822e (Mettler Toledo, Worthington, OH, USA) was carried out to analyze the thermal behavior of raw materials and formulated powders. Thermograms were recorded by placing accurately weighed quantities (3–6 mg weighed with a microbalance MTS Mettler Toledo, Worthington, OH, USA) of each sample in a 40 μL aluminum pan, which was sealed and pierced. The samples underwent one dynamic thermal cycle; they were heated from 25 to 350 °C at a heating rate of 10 °C/min. The instrument automatically determined the baseline as the difference between the latent heat to sensible heat. For each sample, the integrated baseline had been considered and automatically released at the zero point. Dehydration, melting temperature (Tm), and heat of fusion (ΔHm) were measured.

#### 3.3.6. Dissolution/Release Tests

All the tests were carried out in sink conditions, a dissolution system sufficiently diluted thus that the dissolution process was not impeded by saturation of the solution (<35% of extract solubility). HSE solubility and in vitro dissolution/release tests were carried out according to the Farmacopea Ufficiale Italiana, as also reported by Sansone et al., 2013 [53,54].

An excessive amount of HSE was introduced into glass vials containing 5 mL of H_2_O; the samples were stirred and stored at 25 °C for 3 days. After that, samples were centrifuged for 15 min at 3.000 rpm, in order to remove the saturation powder. Supernatants were filtered with 0.45 lm filters, and the concentration of dissolved HSE was determined as lawsonicin equivalents by the HPLC method as described before. The solubility measurements were performed in triplicate.

The determined HSE water solubility (136.74 ± 0.27 mg/L) led to the calculation of the sink conditions for the dissolution/release tests of the unprocessed extract HSE (5 mg/L) and spray-dried formulations (150 mg/L, considering the extract content). Briefly, the test was carried out dissolved samples in 1000 mL of distilled water (sink conditions), using a Sotax AT Smart Apparatus (Basel, CH) and equipped with apparatus 2 of USP 31 for dissolution tests: Paddle, 75 rpm at 37 °C. The duration of the test was 120 min and the samples were taken at 5, 10, 15, 30, 45, 60, 90, and 120 min. Only the mean values were reported (standard deviations < 5%). The amount of the extract dissolved was measured as AAC (Actual Active Content) value was calculated by an HPLC-DAD method, as reported in reference [8]. The standard curve was analyzed using the linear correlation between concentration and peak area integration (regression equation y = 9579.9x − 19.612, r = 0.9999, at 5 concentration levels in the range of 0.01 to 0.00125 mg/mL). All the dissolution/release tests were made in triplicate, each time in 6 vessels; only the mean values were reported (standard deviations < 5%).

### 3.4. Stability Studies

Stability was evaluated under harsh storage conditions, according to the ICH (International Conference Harmonization) guidelines. Glass open vials with 1.5g of each batch were stored for 6 months at 40 ± 2 °C; 75 ± 2% R.H in a climate chamber (Climatic and Thermostatic Chamber, Angelantoni Life Science s.r.l., Perugia, Italy) to investigate the shelf life of the best formulation obtained (Batch-13 and Batch-15).

At time 0 and after 6 months, samples of each batch were collected. Extract content was verified by HPLC method, and morphology and DSC analysis were performed to detect changes in the structure and possible degradation events, according to the previously described methods.

The free-radical scavenging activity of Batch 15 was also evaluated after the spray drying process and after 6 months under accelerated storage conditions using the stable 1,1-diphenyl-2-picrylhydrazyl radical (DPPH), according to our previously reported procedures [55]. Briefly, 1.5 mL of DPPH solution (25 mg/mL in methanol, prepared daily) was added to 0.375 mL of Batch-15 dissolved in water (at 15 mg/mL). The mixtures were kept in the dark for 10 min at room temperature, and the decrease in absorbance was measured at 517 nm against a blank consisting of an equal volume of methanol. α-tocopherol was used as a positive control. The DPPH concentration in the reaction medium was calculated from a calibration curve (range = 5–36 µg/mL) analyzed by linear regression (y = 0.0228x − 0.0350, R^2^ = 0.9999), and EC_50_ (mean effective scavenging concentration) was determined as the concentration (in micrograms per milliliter) of sample necessary to decrease the initial DPPH concentration by 50%. All tests were performed in triplicate. A lower EC_50_ value indicates a stronger antioxidant activity.

#### Hygroscopicity

The hygroscopicity was determined according to the procedure described by Sansone et al. [20] by sampling during the end of the stability test.

Hygroscopicity was gravimetrically calculated according to the formula:

Hygroscopicity (g of H_2_O per g of product) = [(m_3_ − m_0_)/m_3_] × 100 (3)

where m_0_ and m_3_ express the moisture of the samples before (*t*_0_) and after (*t*_6months_) the storage period, respectively.

### 3.5. Cell Viability Assay

Human malignant melanoma (A375 and SK-Mel-28), human cervical cancer (HeLa), and HaCaT (immortalized human normal keratinocytes), and supplements for cell cultures were obtained from Gibco Life Technology Corp. (Thermo Fischer Scientific, Milan, Italy). HaCaT, A375, and HeLa cell lines were grown at 37 °C in Dulbecco’s modified Eagle’s medium containing high glucose supplemented with 10% fetal calf serum, and 100 units/mL each of penicillin and streptomycin, and 2 mmol/L glutamine. Human melanoma (SK-Mel-28) cell line was grown at 37 °C in minimum essential medium (MEM) supplemented with 10% fetal calf serum and 100 units/mL each of penicillin and streptomycin. The potential cytotoxic effect of Batch-15 and the unprocessed extract was investigated by MTT (3-[4,5-dimetiltiazol-2,5-diphenyl-2*H*-tetrazolium bromide]) assay, as reported in reference [56]. To perform the assay, the cells were grown in 96-well plates, in numbers of 7000 per well, and after 24 h were treated with Batch-15 and Batch-12, dissolved directly in the culture medium to obtain the at 15 mg/mL. At the end of treatment, the plates were centrifuged at 1200 rpm for 5 min, the medium was aspirated and added 100 μL of 1 mg/mL MTT to each well, and the plates were kept at 37 °C for the time necessary to the formation of salt formazan (1–3 h depending on cell type). The solution was then removed from each well, and the formazan crystal within the cells was dissolved with 100 µL of DMSO. Absorption at 550 nm for each well was assessed by a Multiskan Spectrum Thermo Electron Corporation Reader. IC_50_ values were calculated from cell viability dose-response curves and defined as the concentration resulting in 50% inhibition of cell survival compared to untreated cells.

### 3.6. Statistical Analysis

All results were shown as mean ± standard deviation of 3 experiments performed in triplicate. Statistical comparison between groups was made using ANOVA followed by the Bonferroni parametric test. Differences were considered significant if *p* < 0.05. The free radical scavenging data were subjected to one-way analysis of variance (ANOVA) followed by and Tukey HSD test (*p* < 0.05), using GraphPad Prism version 7.00 for Windows.

## 4. Conclusions

Hazelnut shells extract has antioxidant and chemopreventive effects on human melanoma and cervical cancer cell lines. However, as all dry plant derivatives, HSE appears as a sticky material, not completely soluble in water and aqueous biological fluids, as well as quite unstable under environmental stress. Our research showed the possibility to successfully transform the raw extract HSE in a spray-dried powder suitable for the final administration in various dosage forms overcoming the stability and bioavailability problems. The tandem polymeric matrix was based on l-proline as a synergic loading carrier, hydroxyethylcellulose/pectin as a coating system, and lecithin as an enhancer of dissolution rate. The development of a multicomponent polymeric matrix, the optimization of the liquid feed preparation method, and the selection of all the spray drying operating parameters led to obtain a stable powder (Batch-15) with high functional content, and both improved water solubility and chemopreventive effects against human cancer cells (A375, SK-Mel-28, and HeLa), easier to be processed for an industrial use with respect to the unprocessed HSE.

## Figures and Tables

**Table 1:** Composition and characteristics of raw materials and spray-dried powders.

Sample	P g/100mL	HEC g/100mL	PEC g/100mL	L_EtOH_ g/100mL	HSE g/100mL	Yield %	AEC ^a^ %	AAC ^b^ %	EE ^c^ %	d_50_ µm (span) ^e^
HSE raw	-	-	-	-	-	-	-	3.16 ± 1.5^d^	-	-
HEC	-	-	-	-	-	-	-	-	-	277.22 (1.60)
P	-	-	-	-	-	-	-	-	-	250.10 (1.71)
PEC	-	-	-	-	-	-	-	-	-	50.53 (1.32)
Batch-3	5.00	0.20	-	-		39.8 ± 4.2 ^d^	-	-	-	-
Batch-9	5.00	0.20	0.50	-	-	45.0 ± 3.1 ^d^	-	-	-	-
Batch-12	5.00	0.20	0.50	0.20	-	50.0 ± 2.0 ^d^	-	-	-	-
Batch-13	5.00	0.20	-	-	0.25	39.8 ± 9.42 ^d^	4.30	3.11 ± 0.90 ^d^	92.10	18.41 (1.63)
Batch-15	5.00	0.20	0.50	0.20	0.25	43.00 ± 3.54 ^d^	3.90	2.10 ± 0.93 ^d^	95.12	3.02 (1.21)

P: Proline; HEC: Hydroxyethylcellulose medium viscosity; PEC: Pectin; L: Lecithin; EtOH: Ethanol; ^a^ Actual Extract Content; ^b^ Actual Active Content; ^c^ Encapsulation Efficiency; ^d^ Average of triplicate analyses ± standard deviation; ^e^ Span value calculated as (d_90_ − d_10_) / d_50_

**Table 2:** Actual Active Content (%) and free-radical scavenging activity of extract before (HSE unprocessed extract) and after microencapsulation process (Batch-15).

	0	6 Months	0	6 Months
Materials	AAC% ^a,b^		DPPH test EC_50_^a,c,d^	
HSE Unprocessed extract	3.16 ± 0.80	1.15 ± 0.40 *	33.42 ± 1.40	40.04 ± 2.11 *
Batch-15	2.10 ± 0.40	2.00 ± 0.60	33.20 ± 0.61	32.80 ±1.01
α-tocopherol ^e^			10.1 ± 1.32	10.12 ± 1.20

^a^ One-way analysis of variance (ANOVA) followed by and Tukey HSD test; means ± SD, * *p* <0.05; ^b^ Actual Active Content (AAC): Content of lawsonicin calculated by HPLC-DAD; ^c^ EC_50_ ± standard deviation (data from three experiments in triplicate); ^d^ In a unit of µg of HSE or Batch-15/mL; ^e^ Positive control of the DPPH test

**Table 3:** MTT assay of raw extract (HSE) and optimized batch (Batch-15).

Cell Line ^a^	IC_50_ HSE Raw mg/mL^b^	IC_50_ Batch-15 mg/mL ^c^
HaCaT	0.500 ± 0.731 ^d^	>15 ^d^
SK-Mel-28	0.459 ± 0.831 ^d^	10.550 ± 3.010
A375	0.584 ± 0.900 ^d^	4.550 ± 1.240 ^d^
HeLa	0.526 ± 0.890 ^d^	8.070 ± 2.150 ^d^

**^a^** A375 and SK-Mel-28, melanoma cells; HeLa, cervical cancer cells; HaCaT immortalized human keratinocytes; **^b^** IC_50_, required concentration of extract to inhibit cell proliferation by 50%; **^c^** IC_50_, concentration of Batch-15 required to inhibit cell proliferation by 50% expressed as mg/mL; in this case, the result is depending on the Actual Extract Content (AEC, 3.9% in Batch-15); ^d^ IC_50_ ± standard deviation (data from three experiments in triplicate).

## Supplementary Materials

Supplementary Materials are available online.
