# Supplementary material for: Design and Development of Spray-Dried Microsystems to Improve Technological and Functional Properties of Bioactive Compounds from Hazelnut Shells
Source: Molecules. 2020 Mar 11;25(6):1273. doi: 10.3390/molecules25061273 (PMC7144004; doi:10.3390/molecules25061273)
Supplement: Supplementary file 1 [file molecules-25-01273-s001.pdf]

This supplementary material contains an extended version of the applied eperimental design plan developed to produce a micrparticulate powder form by spray drying. Authors supply a deepened description of multi-step polymeric matrix development and the relevant critical issues addressed during the laboratory work.

Article

Design and Development of Spray Dried Microsystems to Improve Technological and Functional Properties of bioactive compounds from Hazelnut Shells

Tiziana Esposito, Teresa Mencherini*, Pasquale Del Gaudio, Giulia Auriemma, Silvia Franceschelli, Patrizia Picerno, Rita P. Aquino and Francesca Sansone*

Department of Pharmacy, University of Salerno, Via Giovanni Paolo II, 132, 84084, Fisciano (SA), Italy

* Corresponding authors: Teresa Mencherini (tmencherini@unisa.it); Francesca Sansone (fsansone@unisa.it)

Abstract: An extract obtained from hazelnut shells by-products (HSE) has antioxidant and chemopreventive effects on human melanoma and cervical cancer cell lines, inducing apoptosis by caspase-3 activation. A clinical translation is limited by poor water solubility and low bioavailability. Dried plant extracts often show critical characteristics such as sticky/gummy appearance, unpleasant smell, and instability involving practical difficulties in processing for industrial use. A spray drying method has been applied to transform raw HSE in a microparticulate powder. The biopolymeric matrix was based on L-proline as loading carrier, hydroxyethylcellulose in combination with pectin as coating polymers; lecithin and ethanol were used as solubility enhancers. A Hot-Cold-Hot method was selected to prepare the liquid feed. The so prepared powder showed good technological properties (solid state, particle dimensions, morphology, and water dissolution rate), stability, and unchanged chemopreventive effects with respect to the unprocessed HSE.

Keywords: Hazelnut Shells by-product Extract; Spray Dried Microsystems; Multicomponent-based matrix; long-term stability; improvement of chemopreventive effect

S.1 Microencapsulation process

S.1.1 Blank powders

The viscosity is a limiting parameter to process a liquid feed by the spray drying technique [1,2]. A too high viscosity could obstruct the atomizer nozzle, causing also incomplete solvent evaporation, and low process yields also negatively affecting the particle morphology [3]. Thus, a series of pilot experiments were conducted to determine the appropriate HEC concentration giving a liquid feed compatible in viscosity with the spray drying process (Table S1).

Table S1. Results of HEC viscosity in water at different concentrations.

HEC CONCENTRATION % (w/v)	Relative VISCOSITY* (cP)	Instrumental reliability value**
0.1	68.31	79.51
0.2	75.56	98.42
0.3	81.42	94.44
0.5	81.80	95.71
1.0	92.53	22.00

*** at 60 sec**

**** valid range 18 – 100**

The best instrumental processability was obtained using the 0.1% (w/v) concentration of HEC. However, it was also possible to use HEC concentrations up to 0.3%.

The technological studies start with the design and development of a tandem system polymeric matrix processable by spray drying technique. The matrix system was designed to be able to both efficiently load the polar extract HSE and transform it into a stable functional microparticulate powder useful as a therapeutic ingredient.

More than 45 pilot experiments (blank and loaded powders, Tables S2 and S3) were carried out to find the optimum of liquid feed in term of polymer concentrations and weight polymer ratios with respect to the process yields, loading ability, and resultant powders technological characteristics. As an example, only 12 batches of blank powders and 3 batches of loaded ones (numbered from #1 to 12 and from #13 to 15, respectively) have been selected and reported in the Tables **S**2 and S3, as demonstrative samples of the taken experimental steps.

Table S2. Composition and characteristics of blank powders (Batches 1-12).

Batch number #	HEC g/100mL	P g/100mL	PEC g/100mL	L_{EtOH} g/100mL	Preparation method [a]	Process Yield%
1	0.1	5.0	-		H-C	35.8±5.4 [b]
2	0.1	2.5	-		H-C	34.8±2.3
**3**	**0.2**	**5.0**			**H-C**	**39.8±4.2**
4	0.2	2.5	-		H-C	23.7±3.4
5	0.3	5.0	-		H-C	36.6±3.4
6	0.3	2.5			H-C	25.3±2.1
7	-	5.0	1.0		H-C	45.3±2.7
8	0.2	5.0	0.25		H-C	21.0±2.3
**9**	**0.2**	**5.0**	**0.5**	**-**	**H-C**	**45.0±3.1**
10	0.2	5.0	1.0		H-C	15.6±2.4
11	0.2	5.0	0.5	0.2	H-C	41.0±1.4
**12**	**0.2**	**5.0**	**0.5**	**0.2**	H-C-H	50.0±2.0

The coloured lines represent the most relevant batches
P: proline; HEC: hydroxyethylcellulose medium viscosity; PEC: pectin; L: lecithin; EtOH: ethanol
[a] H-C = Hot/Cold; H-C-H = Hot/Cold/Hot method
[b] Average of triplicate analyses ± standard deviation

As expected, due to the liquid feed viscosity, the batches contained a higher amount of HEC (0.3%, #5 and #6, Table S2) resulted in low process yield and sticky materials with particles not well defined (Figure S1c).

Preliminary satisfactory results in terms of matrix processability were obtained with a HEC and P concentration of 0.1% and 2.5% w/v (Batch-2), respectively. The doubling of concentrations, (HEC 0.2% and P 5.0%, Batch-3, Table S3) led to a slight increase in yield due to a better interaction between the carriers, also reflecting in an improvement in particle formation (Figure S1e).

Anyway, in Batch-3 (Figure S1e) some particles have fractures and clusters on the surface indicating that a coating with HEC alone was not able to completely form the particles. Normally, the morphology of P shows a needle crystalline form [4] (Figure S1a). After the spray drying process, P alone did not lose its crystalline state (Figure S1b); In Batch-3, P interacts with HEC under the spray drying process, involving in a spherical agglomeration process, without totally lose its crystalline state (Figure S1e)[5].

With the aim to both promote the particle formation and reduce the crystalline surface, also increasing process yield, high methoxylated pectin from citrus (PEC) was selected as additional coating polymer, separately included in the basic polymeric matrix. The presence of crystals can fracture the outer surface of the particles and promote the release of the extract. This is a cause of instability because the extract is exposed and therefore subject to degradation. In previous work, PEC has proved suitable to stabilize maltodextrins as a carrier of sticky plant extracts [3].

In order to verify the compatibility between P and PEC, a blank batch (Batch-7) was produced employed P and PEC at 5% and 1%, respectively (Figure S1f, Table S2).

As shown in Figure S1f, the addition of PEC as coating copolymer improves the spherization of the particles and no aggregates were detected.

Anyway, to obtain a powder also useful for topical application, HEC was kept into the liquid feed formulation at the previously tested concentration of 0.2% w/v. To avoid processability problems due to the high viscosity, PEC concentration was kept at 0.25% w/w (Batch-8). In these conditions, it was obtained a decrease in process

yield (21.0 %, Table S2) and the produced particles (Batch-8, Table S2) showed crystalline aggregates on the surface and uncompleted spherization. Thus, the percentage of PEC was tested in formulation up to 1% w/v. The strategy resulted best effective at the 0.5% PEC concentration (Batch-9) (Figure S1g 45% yield, Table S2). Highest concentrations of PEC, in combination with HEC led to a drastic reduction in process yield, probably caused by an increase in the viscosity of the matrix (Table S2).

Once the polymeric matrix conditions were established, the next step was to load the HSE extract. The dry extract had critical solubility characteristics which made it difficult to dissolve or homogeneously suspend, in the produced aqueous matrix. Therefore, Lecithin (L) as emulsifier dissolved in ethanol (EtOH) as a more compatible organic solvent were used as additional GRAS ingredients [6] in the liquid feed preparation (Batch-11, Table S2). As shown in Table S2, the process yield, albeit slightly, decreased with respect to the Batch-9 (without lecithin). Thus, to enhance the interaction of L and EtOH with the polymeric aqueous phase and promoting the formation of an emulsion, the method preparation was modified in a "Hot-Cold-Hot" (H-C-H) method as described in material and methods section. The formulation of a multi-component matrix requires, for reasons linked to the different characteristics of the ingredients, a multi-step preparation method. The Hot Cold method depends on the solubilization conditions of HEC that needs heating to dissolve in water. Subsequently, the extract needs a lower temperature because it is a thermosensitive substance. The use of the Hot-Cold-Hot method with sonication and high dispersion stirring was necessary to use the Lecithin as stabilizer/emulsifier agent also favoring the introduction of the organic-solvent system phase.

The so prepared liquid feed (Batch-12) reflected in the best obtained result in terms of process yield (50%) and particle morphology (Figure S1h).

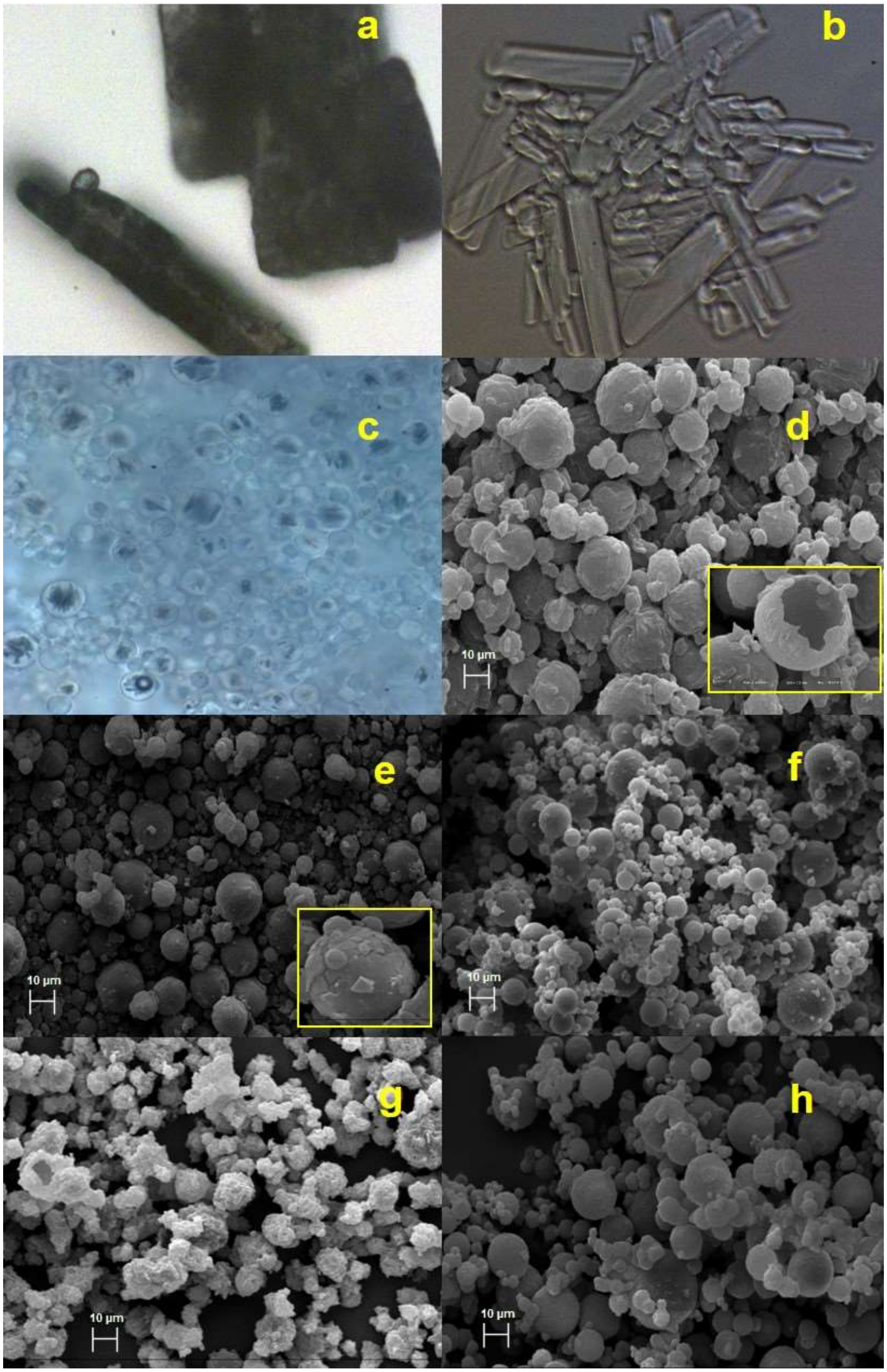

Figure S1. Optical Microscopy images in bright field (**a**) P, raw material, (**b**) P, proline spray drying, (**c**) Batch-5; SEM micrographs (**d**) Batch-1, (**e**) Batch-3, (**f**) Batch-7, (**g**) Batch-9, and (**h**) Batch-12.

S.1.2 HSE-loaded powders

In order to evaluate the influence of HSE on the tandem polymeric system, verifying also the experimental flow design of the blank powders, HSE was separately loaded on the best matrix systems: #3 (only HEC/P) and #12 (HEC/P/PEC/L_{EtOH}) (Table S2) to produce HSE-loaded powders named as batches #13, #14, and #15 (Table S3). Preliminary studies of HSE solubility in polymeric matrices have been conducted up to 0.5 % w/v (Batch-14). Anyway, a so high concentration of HSE negatively affects the process yield (31.5 %, Table S3). The best HSE concentration, compatible with the polymeric system, resulted in the 0.25% w/v (Batch-15, yield 43.0 %).

The composition and main characteristics of the HSE-loaded powders (Batches 13 and 15) had compared each other and to the raw materials (Table S3).

The Actual Active Content (AAC) of both unprocessed and processed extract (AAC-HSE) was determined by an HPLC-DAD method, to verify the percentage of lawsonicin, the most representative neolignan (3.16% w/w) of HSE and thus chosen as a chemical marker. These values led to the calculate the effectively loaded extract (Actual Extract Content, AEC-HSE). AEC- Batch-15 resulted very close to the amount of extract used to prepare the liquid feed (Theoretical Extract Content, TEC-HSE). Consequently, the loading efficiency (LE) value, calculated as the ratio of AEC to TEC, was very satisfying (95.12%, Table S3). Batch-15 was selected as the best batch (% HSE about 5%) instead of Batch-14 (% HSE about 5%) because, the higher concentration of HSE reduces the process yield (from 43 to 31.5%), encapsulation efficiency (from 95.12 to 87.7%), and the Active Content (from 2.10 to 1.98%) although starting from higher amount of loaded extract. Furthermore, the highest % of HSE in Batch-15 negatively affects the morphology and d50 of the microparticles (3.02 μm Batch-15 and 7.21 μm Batch-14).

However, also for optimized powders, the process yield did not exceed 50% probably for the low amount of material sprayed (100 mL) and the loss of the smallest and lightest particles with the exhaust of the spray dryer [7].

Table S3. Composition and characteristics of Batches #13-15 prepared in comparison to the corresponding unprocessed (raw) materials.

Sample	P g/100mL	HEC g/100mL	PEC g/100mL	L_{EtOH} g/100mL	HSE g/100mL	Yield[d] %	AEC [a] %	AAC [b] %	EE[c] %	d_{50} μm (span)[e]
HSE raw	-	-	-	-	-	-	-	3.16± 1.5[d]	-	-
HEC	-	-	-	-	-	-	-	-	-	277.22 (1.60)
P	-	-	-	-	-	-	-	-	-	250.10 (1.71)
PEC	-	-	-	-	-	-	-	-	-	50.53 (1.32)
Batch-13	5.00	0.20	-	-	0.25	39.81±9.42	4.30	3.11± 0.90[f]	92.10	18.41 (1.63)
Batch-14	5.00	0.20	0.50	0.20	0.50	31.50±1.42	3.00	1.98± 0.87[f]	87.70	7.21 (1.00)
Batch-15	5.00	0.20	0.50	0.20	0.25	43.00±3.54	3.90	2.10± 0.93[f]	95.12	3.02 (1.21)

[a]Actual Extract Content
[b]Actual Active Content
[c] Encapsulation Efficiency
[d] Average of triplicate analyses ± standard deviation
[e] Span value calculated as $(d_{90}- d_{10}) /d_{50}$

S.2 Dimensional and morphology analysis

In order to highlight the optimization of the training process, only the results obtained from the technological characterization of batches HSE-loaded powders Batch-13 and Batch-15 (deriving, respectively, from the first and last experimental set) with respect their blank ones (Batch-3 and Batch-12, respectively) and raw materials, are reported and discussed below.

Laser Light scattering (LLs) analysis was carried out to investigate the particle size distribution. As reported in Table S3, Batch-13 and Batch-15 microparticles had different size distribution (d_{50} 18.41 and 3.0 μm) with the lowest mean diameter observed for Batch-15 (Table S3). This difference is due to the right physical interaction between matrix components leading to the rearrangement of P crystals in spherical agglomerates during the spray drying process. The use of the organic lecithin solution to better expose the extract to the aqueous polymeric feed seems to positively affect the interaction of extract with the matrix components, resulting in a reduced dimensional distribution of the obtained particles.

The morphological characterizations of the produced powders were conducted by microscopy analysis (FM and SEM), and the micrographs of obtained samples were compared to HSE and P raw materials. The unprocessed HSE appears as a material in a cluster form with irregular shape and surface (Figure S2a and b) and exploiting red and yellow fluorescence due to the different nature of polyphenols present in the extract. The processed Batch-15 microparticles showed a pale-yellow fluorescent (Figure S2f and g) which was the result of the combination between red/yellow-HSE and blue blank powder as evidence of interaction in forming a homogeneous matrix.

In Batch-13, the extract negatively affected the agglomeration process of P during microparticles formation probably competing with the coating cellulose polymer (Figure S2 c). The powder showed a single cluster zone without a clear microparticles formation (Figure S2 c). FM image showed a not completely loading of the extract that did not interact with the matrix (Figure S2 d).

On the contrary, in Batch-15 (Figure S2 e) spherical, well-formed and completely coated microparticles have been obtained during the spray drying process. Furthermore, the extract is homogeneously distributed and better encapsulated within the microparticles (Figure S2 f and g).

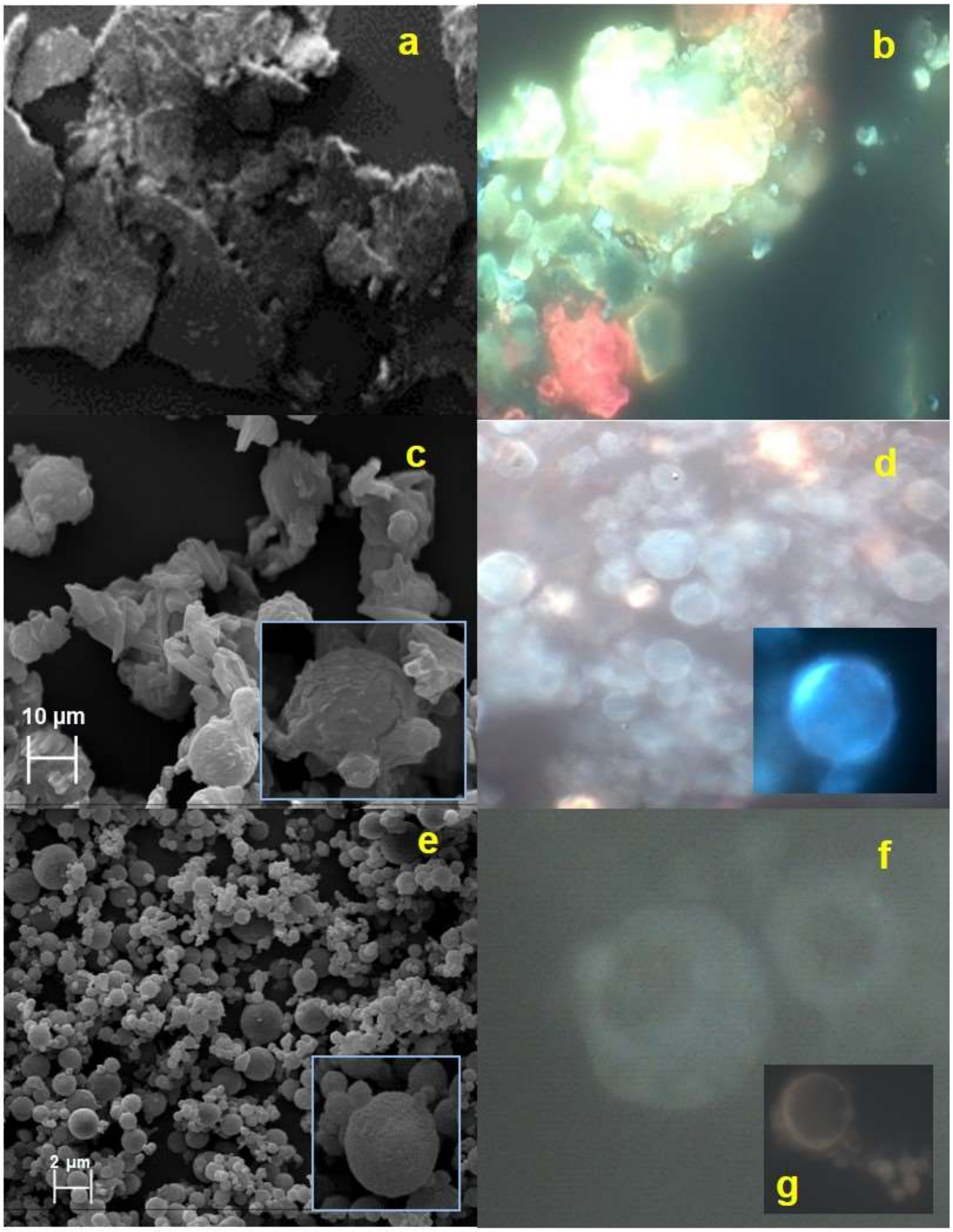

Figure S2. Scan Electron Microscopy (**a**) and Fluorescence Microscopy images (**b**) of HSE raw material in crystalline form; Batch-13 SEM micrographs (c) and FM images (d); Batch-15 SEM micrographs (e) and FM images (f and g).

References

1. O'Sullivan, J.J.; Norwood, E.A.; O'Mahony, J.A.; Kelly, A.L. Atomisation technologies used in spray drying in the dairy industry: A review. *J. Food Eng.* **2019**, *243*, 57–69.
2. Sosnik, A.; Seremeta, K.P. Advantages and challenges of the spray-drying technology for the production of pure drug particles and drug-loaded polymeric carriers. *Adv. Colloid Interface Sci.* **2015**, *223*, 40–54.
3. Sansone, F.; Mencherini, T.; Picerno, P.; D'Amore, M.; Aquino, R.P.; Lauro, M.R. Maltodextrin / pectin microparticles by spray drying as carrier for nutraceutical extracts. *J. Food Eng.* **2011**, *105*, 468–476.
4. Hyung, W.; Kim, Y.; Chung, C.H.; Haam, S. Drowning-out crystallization of l-proline: Effect of anti-solvent composition and processing parameters on crystal size and shape. *Powder Technol.* **2008**, *186*, 137–144.
5. Francia, V.; Martín, L.; Bayly, A.E.; Simmons, M.J.H. Agglomeration during spray drying: Airborne clusters or breakage at the walls? *Chem. Eng. Sci.* **2017**, *162*, 284–299.
6. Chatzidaki, M.D.; Mitsou, E.; Yaghmur, A.; Xenakis, A.; Papadimitriou, V. Formulation and characterization of food-grade microemulsions as carriers of natural phenolic antioxidants. *Colloids Surfaces A Physicochem. Eng. Asp.* **2015**, *483*, 130–136.
7. Panizzon, G.P.; Bueno, F.G.; Ueda-Nakamura, T.; Nakamura, C.V.; Filho, B.P.D. Preparation of spray-dried soy isoflavone-loaded gelatin microspheres for enhancement of dissolution: Formulation, characterization and in vitro evaluation. *Pharmaceutics* **2014**, *6*, 599–615.
